# B Cell Receptor Immunogenetics in B Cell Lymphomas: Immunoglobulin Genes as Key to Ontogeny and Clinical Decision Making

**DOI:** 10.3389/fonc.2020.00067

**Published:** 2020-01-31

**Authors:** Katerina Gemenetzi, Andreas Agathangelidis, Laura Zaragoza-Infante, Electra Sofou, Maria Papaioannou, Anastasia Chatzidimitriou, Kostas Stamatopoulos

**Affiliations:** ^1^Institute of Applied Biosciences, Centre for Research and Technology Hellas, Thessaloniki, Greece; ^2^Hematology Department, University General Hospital of Thessaloniki AHEPA, Thessaloniki, Greece

**Keywords:** chronic lymphocytic leukemia (CLL), mantle cell lymphoma (MCL), germinal center (GC), immunogenetics, ontogeny, patient prognosis

## Abstract

The clonotypic B cell receptor immunoglobulin (BcR IG) plays a seminal role in B cell lymphoma development and evolution. From a clinical perspective, this view is supported by the remarkable therapeutic efficacy of BcR signaling inhibitors, even among heavily pre-treated, relapsed/refractory patients. This clinical development complements immunogenetic evidence for antigen drive in the natural history of these tumors. Indeed, BcR IG gene repertoire biases have been documented in different B cell lymphoma subtypes, alluding to selection of B cell progenitors that express particular BcR IG. Moreover, distinct entities display imprints of somatic hypermutation within the clonotypic BcR IG gene following patterns that strengthen the argument for antigen selection. Of note, at least in certain B cell lymphomas, the BcR IG genes are intraclonally diversified, likely in a context of ongoing interactions with antigen(s). Moreover, BcR IG gene repertoire profiling suggests that unique immune pathways lead to distinct B cell lymphomas through targeting cells at different stages in the B cell differentiation trajectory (e.g., germinal center B cells in follicular lymphoma, FL). Regarding the implicated antigens, although their precise nature remains to be fully elucidated, immunogenetic analysis has offered important hints by revealing similarities between the BcR IG of particular lymphomas and B cell clones with known antigenic specificity: this has paved the way to functional studies that identified relevant antigenic determinants of classes of structurally similar epitopes. Finally, in certain tumors, most notably chronic lymphocytic leukemia (CLL), immunogenetic analysis has also proven instrumental in accurate patient risk stratification since cases with differing BcR IG gene sequence features follow distinct disease courses and respond differently to particular treatment modalities. Overall, delving into the BcR IG gene sequences emerges as key to understanding B cell lymphoma pathophysiology, refining prognostication and assisting in making educated treatment choices.

## Introduction

### B Cell Differentation

Antigen encounter critically shapes the fate of B cells, essentially kicking off two major possible pathways: B cells can either undergo immediate proliferation and differentiation into short-lived plasma cells, or migrate to secondary lymphoid organs and enter specific structures termed germinal centers (GCs). In the former, B cells express polyreactive, low-affinity B cell receptor immunoglobulin (BcR IG) without relevant support from T cells. In the latter, B cells within the GCs are subjected to affinity maturation in a T cell-dependent manner, resulting in the generation of B cells with high-affinity BcR IG, specific for the selecting antigen. Activated B cells with high affinity BcR IG are selectively propagated and mature into two discrete B cell populations: (i) plasma cells that proliferate fast and secrete high-affinity antibodies; and, (ii) long-lived memory B cells capable of eliciting robust responses in future exposure to the original selecting antigen (1–4).

### Differentiation Pathways of Mature B Cells

Memory B cells circulate in the body as resting lymphocytes until reactivation and can be distinguished from naïve B cells based on a series of distinct features. In brief, memory B cells: (i) have increased lifespan; (ii) are mainly found in areas where antigen encounter takes place; (iii) display differential expression of quiescence factors, costimulatory and anti-apoptotic molecules as well as signal transducers; (iv) are capable, upon reactivation, to proliferate and differentiate into plasma cells rapidly secreting large amounts of antibodies, thus, providing a more efficient immune response; (v) can re-enter the GC and be subjected to further affinity maturation in order to refine antigen binding specificity. At the molecular level, affinity maturation is geared by two independent yet mechanistically related processes affecting the clonotypic BcR IG i.e., somatic hypermutation (SHM) and class switch recombination (CSR) (5–7).

SHM is a highly specific mechanism introducing mostly single nucleotide changes into the rearranged variable genes of the BcR IG at a rate far exceeding mutational activity elsewhere in the genome. Mutations do not occur randomly but instead tend to cluster within the regions forming the antigen binding site, namely the complementarity-determining regions (CDRs), enriched for specific nucleotide motifs that serve as SHM hotspots. Within the GC, a single B cell progenitor generates B cells expressing different variants of its BcR IG that display distinct affinities for the given antigen. Some of the mutation combinations will result in higher affinity BcR IG and the B cells expressing these receptors will become favored for activation and proliferation (8, 9). SHM targets not only the rearranged IG genes but also some off-target genes, including *BCL6*, albeit with lower frequency, and even any heterologous sequence introduced downstream the IGHV gene promoter (10).

CSR is the second mechanism participating in affinity maturation within the GCs and is responsible for the change of the BcR IG isotype. There are five different isotypes, namely IgM, IgG, IgA, IgE, and IgD. Naïve B cells express IgM and/or IgD, whereas after antigen encounter B cells can express IgG, IgA, or IgE. CSR changes the constant domain of the BcR IG heavy chain without any effect on the variable domain, leading to the expression of BcR IG with high affinity for the same antigen yet different effector functions (11–13).

Both SHM and CSR depend on the activity of an enzyme called activation-induced cytidine deaminase (AID). AID deaminates a cytosine (C) turning it into uracil (U), thus causing mutations at C-G pairs. A group of low-fidelity DNA polymerases undertakes the repair of the mismatches. In SHM, the U is recognized as thymidine (T) by the repair mechanisms and the C-G pair is converted to T-adenine (A) pair through RNA-editing mechanisms. In CSR, double-stranded DNA (dsDNA) breaks are introduced at the U-G pairs. Joining and repair are targeted by different repair mechanisms leading to chromosomal recombination events (14–16).

In rare instances, memory B cells lack SHM within the BcR IG. A possible explanation for this phenomenon could be that these cells originate from early GC B cells before the actual onset of SHM (5). That said, there is also evidence that B cell activation and the subsequent generation of memory B cells can occur outside the GC environment. Indeed, a GC-independent but T cell-dependent pathway to memory B cell formation has been described, generating memory B cells with limited if any SHM. Furthermore, depending on the type of antigen, the microenvironment where antigen encounter takes place and the particular cytokine milieu, B cells may even undergo SHM and/or CSR outside the GC, although the range of available classes appears to be restricted (17, 18).

A prime example of B cells operating outside the context of GCs concerns the B cell population that resides within the marginal zone (MZ). MZ B cells provide frontline protection against blood borne pathogens and rapidly respond against encapsulated bacteria by differentiating into antigen-specific plasma cells. Additionally, they are responsible for housekeeping activities, since they recognize and remove senescent cells and other cellular debris. In order to serve these functions, MZ B cells express distinctive BcR IG where their antigen reactivity patterns are reflected in a restricted BcR IG gene repertoire. Of note, at least a fraction of MZ B cells, particularly in the spleen, carry SHM within both their BcR IG genes as well as the *BCL6* gene, highlighting an active SHM mechanism. Furthermore, splenic MZ B cells share phenotypic similarities with memory B cells and display enhanced immune response potential. These similarities led to the hypothesis that splenic MZ cells are either of post-GC origin or derive from an independent differentiation pathway (19–22).

### Cellular Origin of B Cell Lymphomas: Overview

Aberrations at any stage in the differentiation process of mature B cells can lead to uncontrolled proliferation and, ultimately, to the emergence of B cell non-Hodgkin lymphomas (B-NHLs) (23, 24). Antigen experienced B cells, such as GC and memory B cells are widely thought to represent progenitor cells for different types of B-NHL, most notably follicular lymphoma (FL) (25), diffuse large B cell lymphoma (DLBCL) (26, 27), and Burkitt lymphoma (BL) (28–30). A key molecular feature of these lymphomas pertains to the identification of SHM imprints within the variable domain of the clonotypic BcR IG, alluding to antigen exposure. This notion is further supported by the pronounced intraclonal diversification of the IG genes, at least in some of these tumors. One of the most notable examples is FL (31–33), where the analysis of somatic mutations led to the notion that SHM is an ongoing process continuously altering the structure of the clonotypic BcR IG under antigenic pressure.

Along the same lines, the study of the BcR IG expressed by the malignant B cells supported potential reactivity against superantigens, at least for a fraction of BL (34) and DLBCL cases. In more detail, the superantigenic binding motifs for N-acetyllactosamine-containing epitopes and Staphylococcal protein A (SpA) have been found intact in BL cases that carry BcR IGs encoded by the IGHV4-34 gene and IGHV3 subgroup genes (34), respectively. Similar findings have been reported for DLBCL cases utilizing the IGHV4-34 gene (35).

Chronic stimulation of the BcR IG by microbial antigens or autoantigens can promote the expansion and progression of malignant B cells. This is amply exemplified by gastric MALT lymphoma that is strongly associated with chronic infection by *Helicobacter pylori* (36). Similar links to pathogens have been identified for extranodal MZ lymphomas (ENMZL) of different tissues, such as ocular adnexa MZ lymphoma and cutaneous MZ lymphoma, which have been associated with infections by *Chlamydia psitacci* and *Borrelia burgdorferi*, respectively (37). Moreover, ENMZL of the salivary and the thyroid glands have been linked to continuous triggering by autoantigens responsible for Sjögren's syndrome and Hashimoto thyroiditis, respectively (38). Interestingly, BcR IG gene repertoire restrictions and distinctive SHM patterns characterize ENMZL as well, albeit with significantly different IG gene distributions depending on the primary site of involvement, indicating distinct antigen exposure histories (39, 40). Extensive immunogenetic profiling of MZ lymphomas and cross-comparison to other B-NHLs has also documented the existence of rare public BcR IG stereotypes shared by different entities. This finding raises the intriguing possibility that common immune mechanisms triggered by pathogens may underlie the ontogeny of diverse B lymphoproliferations likely due to targeting versatile progenitor B cells in particular microenvironments, including the GC but, perhaps, also extrafollicular sites (39).

The aforementioned examples constitute proof-of-concept about antigen-driven lymphomagenesis, while also highlighting the critical role of affinity maturation processes, particularly SHM, in B-NHL development and evolution. In these examples, the putative cell of origin can be pinpointed with some degree of certainty to a GC or post-GC cell. In the sections that follow, we will start with FL as a reference case for GC-originating lymphomas and then focus on certain other lymphoproliferative entities where the putative cell(s) of origin still remain(s) elusive. We will present the immunogenetic evidence that have assisted in gradually revealing the implicated ontogenetic processes while also acquiring an ever-increasing role in prognostication and clinical decision-making at large.

### Follicular Lymphoma

Follicular lymphoma (FL), the second most common nodal lymphoma, is generally considered as a prototype of indolent lymphomas displaying a clinical course that is characterized by slow progression and high response rates to therapy (41). However, a significant fraction of patients with FL eventually develop resistant disease, while in almost half of cases the original indolent disease transforms into an aggressive subtype, such as DLBCL (42, 43).

It is widely established that FL arises in germinal centers, hence maintaining features of normal GCs. In more detail, FL cells form follicles surrounded by non-malignant antigen presenting cells (including T cells, dendritic cells and macrophages) (44); express GC surface markers such as BCL6 and CD10 (44); and, display a gene expression signature that is similar to that of centrocytes and/or centroblasts (26).

Most patients with FL (around 85%) carry the t(14;18) (q32;q21) chromosomal translocation, which constitutes a hallmark of this lymphoma (45). In specific, this translocation involves the *bcl-2* gene (B cell leukemia/lymphoma 2) and the IgH (immunoglobulin heavy chain) gene locus, leading to the overexpression of the BCL2 protein that prevents cells from undergoing apoptosis. The increased frequency of t(14;18) in FL together with its presence at diagnosis support its consideration as the initial oncogenetic hit during the development of FL (41).

In regard to the timing of the t(14;18) in the natural history of FL, it was initially accepted that it takes place early in B cell development, during the initial phase of the V(D)J recombination process that involves the rearrangement between a IGHD and a IGHJ gene. However, the analysis of *BCL2*-IGH junctions (46, 47) provided evidence that the translocation event can also occur at a more advanced stage of the V(D)J recombination process, when an IGHV gene recombines with the assembled IGHD-IGHJ gene complex.

An alternate scenario (41) involved naive B cells carrying the t(14;18). These, previously unselected cells, would exit the bone marrow and move to secondary lymphoid tissues, where they would undergo the GC reaction. The constitutive expression of BCL2, not normally expressed in the GC microenvironemnt, could offer a selective advantage to these cells by protecting them from apoptosis (48). Finally, FL progenitor cells would acquire secondary genetic lesions due to activation induced cytidine deaminase (AID) activity (49) and transform to FL cells. This scenario of FL ontogenesis was challenged by the observation that cells similar to FL progenitor cells can be detected in the blood of healthy individuals who did not develop FL (50–52). Moreover, recent studies showed that most of the FL progenitor cells have probably undergone some kind of selection since they were not naive B cells, but rather IgD^+^CD27^+^ (or IgM^+^CD27^+^) memory cells that are GC-experienced (53, 54). Similar to FL cells, these FL-like cells in healthy individuals frequently show evidence of class-switch recombination of the translocated IGH allele, whereas the functional allele encodes a surface IgD (or IgM). The role of these FL-like cells in the pathogenesis of FL remains unknown.

Nonetheless, FL progenitor cells do exist and have a far more complicated genetic background than previously thought, as shown in 2 case reports where both the donor and the recipient developed FL after allogeneic hematopoietic cell transplantation (55, 56). In the most recent study (56), the t(14;18) was detected in the donor sample along with 14 of the 15 gene somatic mutations that were present in the tumors from both the donor and the recipient, yet at a much lower frequency.

Our understanding of the origin of FL is further complicated by an entity called FL *in situ*, which has been proposed as a true precursor lymphoma state (57, 58). However, this entity needs to be further studied in order to gain better insight into its natural history and relation, if any, to early stage FL.

#### Supraphysiological N-Glycosylation of the Clonotypic Immunoglobulin and Cellular Activation in Follicular Lymphoma

Immunogenetic studies in FL reported an unbiased IGHV gene repertoire. In the vast majority of FL cases, the rearranged IGHV genes carry imprints of SHM, with most mutations located within the CDRs (59–61). The analysis of SHM patterns in FL also revealed pronounced intraclonal diversification, indicating that the lymphoma cells further diversify their IG genes through ongoing SHM (32, 62). IG light chain genes also display imprints of SHM, albeit to a lesser degree than their partner heavy chains (63) (Table 1).

**Table 1:** Overview of the immunogenetic profiles of CLL and MCL.

**Immunogenetic characteristics**	**FL**	**CLL**	**MCL**
IGHV gene repertoire	No major biases.	Disease-specific biases (dominance of IGHV1-69, IGHV3-7, and IGHV4-34).	Disease-specific biases (dominance of IGHV3-21, IGHV4-34, IGHV1-8, IGHV3-23)ref.
SHM status	Most cases carry somatic mutations in the heavy chains. Very few mutations were identified in the light chains. Mutations clustered within the CDRs. A pattern of ongoing mutations was observed in a significant fraction of cases.	Significant SHM imprint (GI < 98%) in more than 50% of cases. Disease-specific, recurrent SHMs at the individual IGHV gene level. Important prognostic implications.	SHM (GI < 100%) present in 70% of cases. Specific SHM targeting at the individual IGHV gene level. No solid correlations between SHM status and patient prognosis.
BcR IG stereotypy	Not found.	Stereotyped subsets account for around 30% of cases.	Stereotyped subsets account for >10% of cases utilizing mainly the IGHV3-21 or and IGHV4-34 genes.

*FL, folicullar lymphoma; CLL, chronic lymphocytic leukemia; MCL, mantle cell lymphoma; IGHV, immunoglobulin heavy variable; SHM, somatic hypermutation; BcR IG, B cell receptor immunoglobulin*.

A striking feature of the BcR IG in FL concerns the unusually high incidence of novel N-glycosylation sites (Asn-X-Ser/Thr) introduced by SHM mostly in the CDRs of the heavy chains and less frequently in the light chains (64). BcR IG are known to be variably glycosylated, most commonly within the constant region of the molecule. However, the variable region may also be glycosylated and this modification is known to affect the process of antigen recognition and binding (65). In FL, these novel N-glycosylation sites are present in essentially all cases at diagnosis (64, 66) indicating that they are introduced at early stages of disease ontogeny. These modifications were not frequently present in normal B cells, suggesting their potential relevance for FL pathogenesis (64, 67).

Analysis of the added glycans in the BcR IG from FL revealed that they concern oligomannoses in the variable regions but complex sugars in the constant regions (68). These glycans interact with mannose-specific lectins, especially with dendritic cell-specific intercellular adhesion molecule-3-grabbing non-integrin (DC-SIGN) expressed by dendritic cells, macrophages and lymphatic endothelial cells. Lectin binding to FL triggers persistent activating signals leading to intracellular Ca^2+^ increase, sustained phosphorylation of the SYK, AKT, PLCγ2, and ERK1/2 kinases downstream the BcR, and increased expression of cMYC (69–71). This finding suggests that interactions between FL cells and the tumor microenvironment affect and promote tumor progression.

Another immunogenetic feature that could likely impact on binding to lectins concerns the IG isotype, however the published evidence remains controversial. In a recent study (70), activation by DC-SIGN occurred in FL cases of either the IgM or the IgG isotype, whereas in another study (71) only IgM^+^ FL cells could respond to lectin binding. IgG^+^ FL cases carry BcR IG that are more commonly auto-reactive compared to those expressed by IgM^+^ FL cases (72). Hence, arguably, although IgG^+^ FLs contain inserted N-glycosylation sites as in the case of IgM^+^ FLs, lectin-mediated BcR IG triggering has a greater effect on non-auto-reactive FLs (IgM^+^). That said, additional studies are required in order to fully elucidate the role, if any, of lectin-induced BcR IG activation in the natural history of IgG^+^ FL.

### Chronic Lymphocytic Leukemia

Chronic lymphocytic leukemia (CLL) is a chronic B cell malignancy, representing 30–40% of all adult leukemias (73). It is a disease of aged individuals with unknown etiology and variable clinical course, ranging from very indolent to rather aggressive, intricately linked to and likely reflecting the underlying biological diversity. Indeed, CLL is characterized by a complex biological landscape. The combined effect of cell-intrinsic aberrations and microenvironmental triggering underlies the characteristic resistance to apoptosis, while also promoting cell proliferation, ultimately driving disease progression (74, 75).

#### CLL Clones Express a Restricted B Cell Receptor Immunoglobulin Gene Repertoire

Immunogenetic analysis has been at the forefront of CLL research for more than two decades, offering robust evidence that the clonotypic BcR IG engages in specific recognition of and selection by (auto)antigen. This process likely shapes clonal behavior and eventual clinical outcome. It all started in the 1990s when studies reported restricted usage of certain IGHV genes (IGHV1-69, IGHV3-7, IGHV4-34) by CLL cells and, in parallel, documented the existence of SHM patterns consistent with antigen selection in a substantial fraction of cases (76, 77). These findings were subsequently confirmed in larger cohorts strongly implying the selection of CLL progenitor cells by a restricted set of antigens (78, 79).

A turning point in CLL research was the demonstration that roughly 50% of cases utilizing the IGHV3-21 gene displayed highly similar heavy variable CDR3 (VH CDR3). These CLL cases also expressed quasi-identical light chains encoded by the IGLV3-21 gene (80). Clearly at odds with serendipity, this remarkable restriction argued for antigenic pressure leading to the selection of particular features of the clonotypic BcR IG. Soon thereafter, it became apparent that antigen binding site restrictions was a feature of the CLL BcR IG repertoire beyond IGHV3-21 expressing cases: indeed, a sizeable fraction of unrelated CLL patients were assigned to subsets characterized by highly similar, “stereotyped” VH CDR3 sequences. On these grounds, it was reasonably suggested that BcR IGs belonging to the same stereotyped subset were selected by a restricted range of antigenic epitopes (81–88).

Delving deep into this phenomenon in increasingly populated CLL cohorts (89, 90) revealed that BcR IG stereotypes collectively accounted for almost one-third of the BcR IG repertoire in CLL and could be classified into a large number of subsets, ranging in size from only a pair to hundreds of cases (“major” subsets) (Table 1). Inevitably, it became relevant to address the issue of whether stereotyped BcR IGs were exclusive to CLL or could also be found in other B-NHLs and/or other, non-malignant entities. Cross-entity comparisons identified only a few shared homologous VH CDR3, thus revealing that the majority of stereotyped BcR IGs are “CLL-biased.” The few BcR IG sequence matches concerned sequences from autoreactive B cell clones, as well as sequences from diverse B cell lymphoproliferative disorders directly or indirectly linked to infections by certain pathogens (e.g., the *hepatitis C* virus in the case of CLL stereotyped subset #13) (91). These observations supported the notion that, occasionally, common progenitors may give rise to distinct pathologies. Although the implicated mechanisms remain obscure, this observation underscores the versatility of B cells while also questioning the relevance of “straightforward” one-to-one matching of normal B cell subpopulations and B-NHLs.

#### Immunogenetics and the Ontogeny of CLL

**Figure 1:**
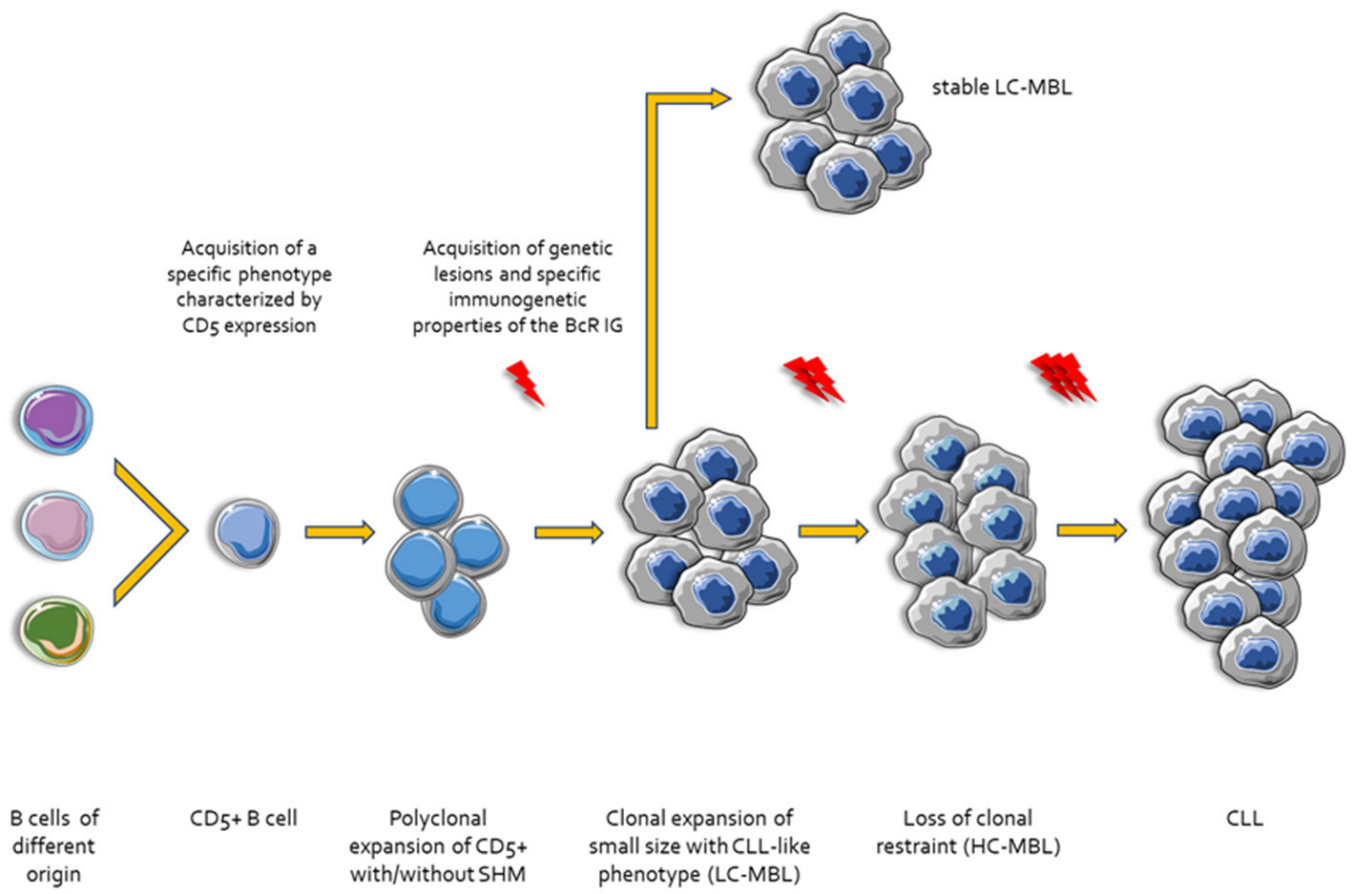
Ontogenetic scenarios for CLL. Different types of B cells stimulated by microenvironmental triggers acquire a specific phenotype characterized by the expression of CD5. Then, CD5+ B cells undergo clonal expansion with or without SHM and CSR. In this step, these small size clonal expansions may acquire genetic lesions display specific CLL-like immunogenetic characteristics and are referred to as Low Count-Monoclonal B-cell Lymphocytosis (LC-MBL). After acquisition of additional genetic and epigenetic changes and/or molecular characteristics of the BcR IG the clones lose the clonal restraint (High Count-MBL, HC-MBL). Finally, HC-MBL cases progress to overt CLL.

The presence of SHM within the clonotypic IGHV genes of the malignant clones dichotomize CLL into two broad categories with postulated distinct origin: (i) cases with few or no SHM (unmutated CLL, U-CLL) purportedly originating from B cells at a point of differentiation prior to the accumulation of high levels of SHM in the context of a GC reaction (naïve B cells?); and, (ii) cases with a heavy SHM load (mutated CLL, M-CLL) that could reasonably be thought to derive from B cells antigen-selected in a classic T cell-dependent manner within the GC microenvironment (92). However, despite appearing reasonable, this orderly “binary” theory does not exhaust all potential options and scenarios. One such option could be that at least a fraction of CLL clones might derive from extrafollicular B cells. This concept is grounded on the fact that low-affinity BcR IG with few or no traces of SHM can arise in a T cell-independent manner in the MZ. A similar immunogenetic profile has been reported for memory B cells generated through proliferative expansions early after immunization in a T cell-dependent but GC-independent manner (17) (Figure 1).

Interestingly, CLL monoclonal antibodies (mAbs), especially those with no or limited SHM, display similarities with natural antibodies present in the blood circulation of healthy individuals that recognize non-protein antigenic epitopes common in pathogenic and commensal organisms as well as cellular debris (19, 20). In fact, similar to natural antibodies, recombinant mAbs from U-CLL clones were found to exhibit polyreactivity and low-affinity binding against apoptotic cells and bacteria (93–100). On the other hand, recombinant mAbs from M-CLL clones recognized a more restricted set of antigenic epitopes, yet with higher affinity. Relevant to mention, when mutated mAbs were reverted back to their germline configuration they acquired reactivity against a more extended range of antigens, thus indicating that both U-CLL and M-CLL could derive from autoreactive progenitor B cells (96).

In mice, natural antibodies originate from B-1 cells, a self-renewing CD5^+^ B cell population with a distinctive, highly restricted BcR IG gene repertoire. The existence of a distinct B-1 lineage with unique properties and role in humans is still contested (101, 102). That notwithstanding, the immunogenetic analogy between B-1 cells and stereotyped CLL clones, especially those belonging to U-CLL, cannot be overlooked, prompting speculations that such clones could originate from a (still elusive) population of evolutionarily conserved B cells functionally intermediate between innate and adaptive immune cells. Put differently, BcR IG stereotypy may reflect origin from a particular cell population characterized by inherent invariance that is responsible for highly conserved, “housekeeping” immune functions (90).

An alternative approach to tracing the cellular origin of CLL was to assess AID expression and functionality. Pioneer studies showed that AID was expressed at higher levels and was more active *in vivo* in U-CLL compared to M-CLL (103, 104). More extensive studies demonstrated that, in both CLL mutational subgroups, AID was fully functional in only a small fraction of cells that had recently divided or were actively dividing (105). Moreover, it was reported that *ex vivo* exposure of CLL cells, from both U-CLL and M-CLL, to stimuli mimicking T cell triggering resulted in upregulated expression of AID (105). These intriguing findings challenged a naïve origin for U-CLL, instead supporting that the properties of the corresponding unmutated or minimally mutated BcR IG were optimal for clonal vigor, hence functionally selected.

Additional *in vitro* studies focusing on the signaling capacity of the BcR IG showed differential signaling capacities in U-CLL and M-CLL (106–109). The observed differences in the ability to signal through the BcR IG can be attributed to either differences in the nature and strength of antigenic stimulation or the different cellular origin of U-CLL and M-CLL. In specific, mutated BcR IGs were more often associated with decreased signaling capacity due to desensitization, ultimately leading to anergy. In contrast, U-CLL cases were shown to express more competent BcR IG indicating that antigenic stimulation can promote the survival and growth of the leukemic cells possibly explaining disease aggressiveness (110–112).

Besides classic antigen-driven stimulation, cell-autonomous signaling triggered by BcR IG self-association has been proposed as an alternative mode of activation exclusive for CLL amongst all studied mature B-NHLs. This signaling mode was shown to promote Ca^2+^ influx and nuclear factor-κB target gene transcription without the implication of exogenous antigen (113). Cell-autonomous signaling was also shown to play a role in leukemia development in the EμTCL1 mouse model that represents an established animal model of aggressive CLL (114).

Recently, we investigated the mechanism of cell-autonomous signaling in cases belonging to stereotyped subsets #2 and #4 that represent paradigmatic examples of aggressive and indolent disease, respectively. We documented BcR IG self-association in both subsets, albeit with different, subset-specific patterns of interactions and resultant cell activation status (115). These findings are not only relevant to CLL biology but also offer a novel perspective to BcR antagonism as an potential therapeutic strategy for CLL.

#### Immunogenetics in Clinical Decision-Making

In 1999 two independent studies reported that the SHM status of the clonotypic rearranged IGHV genes has important prognostic implications since U-CLL patients generally experience a significantly more aggressive disease course compared to M-CLL patients (116, 117). In the ensuing years, IGHV gene SHM status emerged as one of the most robust prognostic markers, independent of clinical stage or other biomarkers. More importantly, it remains stable overtime, thus contrasting other well-established prognostic/predictive markers, including genomic aberrations, which are influenced by or reflect disease evolution (118).

Thanks to the evidence amassed over the last 20 years, it is now widely held that the SHM status of the rearranged IGHV gene represents a cornerstone for the development of biologically-grounded prognostic schemes enabling accurate risk stratification in CLL. This notion is supported, amongst others, by the asymmetric distribution of cell-intrinsic aberrations with important prognostic/predictive value (e.g., cytogenetic aberrations and gene mutations) between U-CLL vs. M-CLL (119–121). Characteristic examples concern the adverse-prognostic del(17p) and del(11q) that are significantly enriched in the former, thus contrasting *MYD88* mutations which predominate by far in the latter. Interestingly, even more striking associations between immunogenetic characteristics and oncogenetic aberrations have been reported in CLL subsets. For instance, stereotyped subset #2 exhibits a 45–50% incidence of *SF3B1* mutations as opposed to only 5–10% in generic CLL cohorts (122, 123); moreover, stereotyped subset #8 (IGHV4-39/IGKV1(D)-39), the CLL subgroup with the highest risk for Richter's transformation amongst all CLL (124), exhibits a ~60% incidence of trisomy 12 as opposed to only ~15% in generic CLL cohorts (120). Subsequently, similar observations have been made for other B-NHL as well e.g., significant enrichment of (i) *TNFAIP3* mutations in ocular adnexa lymphoma cases expressing IGHV4-34 BcR IG (125, 126); (ii) *KLF2* mutations in splenic marginal zone lymphoma cases expressing IGHV1-2*04 BcR IG (127–129). These findings indicate that a distinctive signaling capacity shaped by particular BcR IG may favor the acquisition and/or selection of certain ongogenetic hits by as yet undetermined mechanisms.

**Figure 2:**
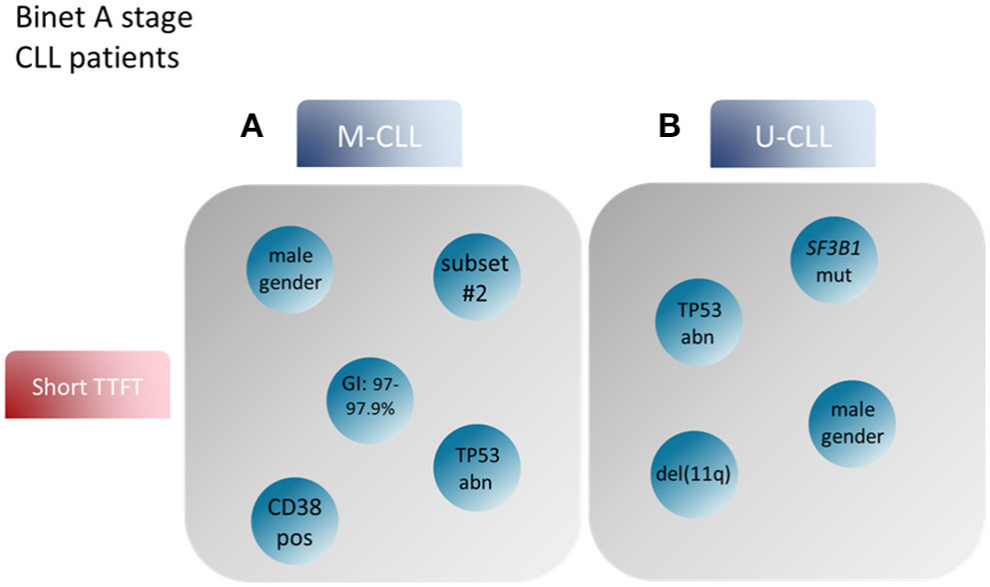
Prognostic factors for CLL patient groups based on the mutational status of the BcR IG. Modified after Baliakas et al. (132). **(A)** Prognostic factors such as male gender, mutational status around the 2% cutoff, TP53abn, +12 and assignment to stereotyped subset #2 correlated with short TTFT in M-CLL. **(B)** On the other hand, male gender, TP53abn, SF3B1 mutations and del(11q) in U-CLL patients were associated with similar, short TTFT (99). BcR IG, B cell receptor immunoglobulin; TTFT, time to first treatment; TP53abn, del(17p) and/or TP53 mutations; +12, trisomy 12; M-CLL, CLL with mutated IGHV genes; U-CLL, CLL with unmutated IGHV genes.

Returning to prognostication, another point to consider concerns the differing relative significance of cell-intrinsic aberrations in U-CLL vs. M-CLL. Hence, trisomy 12 appears to be associated with a rather favorable outcome when present in U-CLL whereas the opposite holds true for M-CLL. A similar observation has been made for *TP53* aberrations [del(17p) and/or *TP53* mutations], where M-CLL patients showed significantly longer time-to-first-tratment (TTFT) and overall survival (OS) compared to U-CLL patients carrying the same genomic aberrations IG (130, 131). Prompted by these observations, more recently, we followed a compartmentalized approach in order to study the prognosis of CLL patients divided into M-CLL and U-CLL and identied distinct factors contributing to disease prognostication in each subgroup (132). In detail, whereas *TP53* aberrations were associated with inferior outcome in both M-CLL and U-CLL, trisomy 12 and/or stereotyped subset #2 membership constituted adverse prognosticators for the former whereas *SF3B1* mutations and del(11q) had the most significant negative impact amongst the latter (132) (Figure 2).

With hindsight, the 1999 publications from the Chiorazzi and Stevenson groups on IGHV gene SHM status and prognostication in CLL represent a true landmark in cancer research due to linking a “physiological” mark of immune maturation with disease outcome (116, 117). The immediate, direct effect was to spur an intense study of IG genes: thus, a research activity reserved for specialized labs became a test for routine diagnostic labs. This led to the amassment of IG gene sequences from thousands of patients, a true treasure trove of information with not only prognostic but also biological relevance. The late, indirect effect was a major contribution toward a paradigm change in CLL treatment brought about by the advent of BcR signaling inhibitors (BCRi) i.e., ibrutinib, a BTK inhibitor, and idelalisib, a PI3Kδ inhibitor (133, 134).

Additional evidence strengthening the clinical importance of immunogenetics in CLL came about more recently from studies showing that IGHV gene SHM status was also strongly associated with the clinical response to chemoimmunotherapy (more particularly the fludarabine cyclophosphamide rituximab regimen, FCR). In more detail, the duration of 1st complete remission (CR) was significantly shorter in U-CLL compared to M-CLL, highlighting that theurapeutic interventions should be tailored to the SHM status (135). Of note, such effect was not seen with ibrutinib as 1st line treatment, where this agent proved equally effective across both U-CLL and M-CLL (133). On this evidence, the 2018 iwCLL guidelines explicitly state that this biomarker should be assessed for all CLL patients in both general practice and clinical trials (136).

Is everything settled when it comes to immunogenetics and clinical decision making? For a number of reasons, the answer appears to be no. First, the distinction into U-CLL vs. M-CLL relies on the use of a 98% cut-off value of identity between the clonotypic rearranged gene and its closest germline counterpart. Although this cut-off still holds, caution is warranted for M-CLL cases with IGHV germline identity close to the 98% value (i.e., 97–97.9%) for which the term “IG-borderline” was coined. These cases do not appear to represent a homogeneous group with intermediate prognosis but rather a mix of indolent and aggressive cases, clearly indicating the need for further study (137). Second, ample evidence now exists that subgroups of patients within each mutational group (i.e., M-CLL and U-CLL) may deviate from the expected “norm” for that group: the most compelling case concerns stereotyped subsets.

Indeed, the study of major CLL stereotyped subsets has revealed that similarities between cases in a given subset extend beyond immunogenetic and other biological characteristics of the malignant clones (genomic aberrations, epigenomic status, BcR IG 3D conformation and signaling capacity) to disease course and outcome (108, 115, 120, 138–141). A most characteristic example concerns stereotyped subsets #2 (IGHV3-21/IGLV3-21 BcR IG) and #4 (IGHV4-34/IGKV2-30 BcR IG). Though both concern mostly M-CLL, the former displays a very aggressive clinical course similar to that of patients with *TP53* aberrations, despite rarely carrying such lesions, thus sharply contrasting subset #4 that has emerged as the prototype of indolent CLL (120). Therefore, BcR IG stereotypy may assist meaningfully in refining prognostication in CLL beyond the binary M-CLL vs. U-CLL distinction, although more evidence is essential before integrating this information into clinical decision making, a view also shared by the recent iwCLL guidelines.

### Mantle Cell Lymphoma

Mantle cell lymphoma (MCL) is an aggressive lymphoma representing 5–10% of all B-NHL. In sharp contrast to the remarkably heterogeneous profile of genomic aberrations in CLL, MCL is characterized by the almost ubiquitous presence of the t(11;14) (q13;q32) chromosomal translocation underlying the formation of the IGH/CCND1 fusion gene and, ultimately, leading to cyclin D1 overexpression, a true pathologic hallmark of MCL (142, 143).

Traditionally, MCL has been associated with a rather aggressive clinical course. However, MCL patients with specific clinical characteristics, such as those with non-nodal disease presentation, tend to follow a more indolent course of the disease, characterized by prolonged TTFT and PFS as well as excellent OS (144). Until recently, the reason for this divergent clinical behavior, especially in view of the quite consistent genomic landscape, has remained elusive.

#### Immunogenetics in MCL: An Indelible Imprint of Antigen Selection

Immunogenetic studies from the early 2000s reported overexpression of certain IGHV genes in MCL, alluding to functional selection (145–150). However, due to the small sample size of these studies, definitive conclusions could not be drawn until almost a decade later when the analysis of a large MCL series (>800 cases) provided compelling evidence implicating antigen involvement in disease ontogeny (151). In more detail, just four IGHV genes, namely IGHV3-21, IGHV4-34, IGHV1-8, and IGHV3-23, collectively accounted for almost 50% of the total repertoire. SHM was present in the clonotypic rearranged IGHV genes of ~70% of MCL cases with patterns indicative of a post-GC derivation; moreover, in analogy to CLL, asymmetries were noted regarding IGHV gene usage in subgroups of MCL with distinct SHM status. Last but not least, stereotyped BcR IG were documented in MCL based on the presence of specific gene associations and shared amino acid motifs within the VH CDR3 region. Stereotyped subsets in MCL represented almost 10% of all cases, the vast majority utilizing either the IGHV3-21 or the IGHV4-34 gene (Table 1).

Not paradoxically, comparisons were made to CLL, especially regarding BcR IG stereotypes and SHM patterns, revealing disease-biased profiles. Furthermore, stereotyped BcR IG utilizing the same IGHV gene (e.g., IGHV3-21 or IGHV4-34) clearly differed in MCL vs. CLL, hence could be safely considered as disease-specific (151). Furthermore, several recurrent SHM present in rearrangements of a given IGHV gene were found to be “MCL-biased” since they were either under-represented or completely absent in other B cell malignancies. Overall, these findings constituted a unique profile arguing compellingly for antigen selection in MCL ontogeny (151).

Prompted by the CLL case, many studies have investigated whether IGHV gene SHM status may serve as a prognosticator also in MCL, however the obtained results were not always consistent (146–149). Still, a tendency of MCL patients with a heavier SHM load to experience a more indolent disease was evident. These patients presented more frequently with early-stage disease, absence of bone marrow infiltration and non-nodal disease and, moreover, were characterized by lower relapse rates and longer relapse-free survival (149, 152) (Table 1).

#### How Many Ontogenetic Pathways to MCL?

Considering the evidence presented above, one could draw a plausible ontogenetic scenario for MCL whereby cases with unmutated BcR IG might derive from a pre-GC naïve B cell, whereas those with mutated BcR IG might derive from an antigen-experienced, post-GC B cell (142). In line with this, SOX11-positive, BcR IG-unmutated clones had a gene expression profile (GEP) similar to that of naïve B cells, whereas the GEP of SOX11-negative, BcR IG-mutated patients was close to that of memory B cells (152).

**Figure 3:**
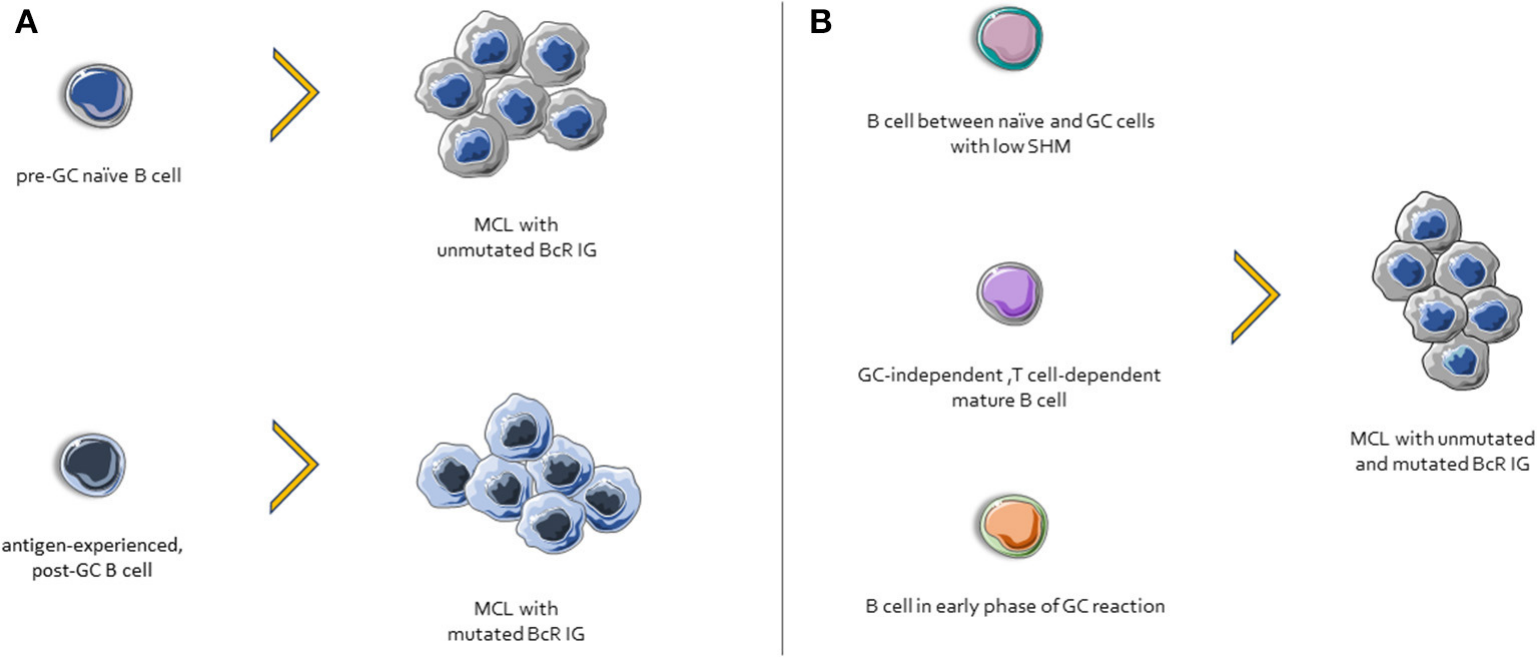
Possible scenarios for MCL ontogenesis. **(A)** Cases with unmutated BcR IG derive from naïve B cells, whereas those with mutated BcR IG might derive from an antigen-experienced, post-GC B cells. **(B)** Other possible MCL progenitor cells include: (i) a normal B cell subpopulation between naïve and GC cells, characterized by few SHMs, (ii) cells that differentiated in a GC-independent but T cell-dependent microenvironment, and (iii) B cells from early phases of GC reactions.

However, other evidence questions this scenario. First, BcR IG stereotypes, a telltale sign of selection, were found even in cases lacking any SHM within the clonotypic IGHV genes (151). Second, almost all MCL cases express AID, with significantly higher levels amongst those with unmutated BcR IG (153), whereas a fraction of cases exhibit ongoing CSR *in vivo* (154). Third, MCL clones, regardless their SHM status, respond favorably to the BTK inhibitor ibrutinib (155). Hence, alternative ontogenetic scenarios can be envisioned (156), including, amongst others, a normal B cell subpopulation intermediate between naïve and GC cells, with a low impact of SHM, and an IgM^+^IgD^+^CD27^−^CD23^−^CD5^+^CD10^−^ phenotype (157); B cells maturing in GC-independent but T cell-dependent pathways; B cells participating in early phases of GC reactions, prior to class switching etc. (Figure 3).

Amidst all this uncertainty, one thing is certain: naivety does not become MCL!

## Concluding Remarks

B cell maturation is a highly complex process where even small derogations can pave the way to the development of malignancy. Critical for the neoplastic transformation of mature B cells is the communication with the tumor microenvironment, more specifically, antigenic stimulation. The key role of antigens in the onset and evolution of mature B cell lymphomas has been corroborated by ever-growing evidence from different, complementary research fields. Immunogenetics has been at the forefront of such endeavors and is reasonably anticipated to hold its leading role in the years to come.

## Author Contributions

KG, AA, LZ-I, and ES wrote the manuscript. MP, AC, and KS supervised and wrote the manuscript.

### Conflict of Interest

The authors declare that the research was conducted in the absence of any commercial or financial relationships that could be construed as a potential conflict of interest.
